# Chemical Profiling and Quantification of Potential Bioactive Components in Gandouling Pill by Ultra-High Performance Liquid Chromatography Coupled with Diode Array Detector/Quadruple-Qrbitrap Mass Spectrometry

**DOI:** 10.3390/molecules27238247

**Published:** 2022-11-26

**Authors:** Yue Yang, Wenjie Hao, Yulong Yang, Shijie Zhang, Han Wang, Meixia Wang, Ting Dong, Zhanpeng Shang, Wenming Yang

**Affiliations:** 1 Graduate School of Anhui University of Chinese Medicine, Hefei 230038, China; 2 The First Affifiliated Hospital of Anhui University of Chinese Medicine, Hefei 230022, China; 3 School of Pharmaceutical Sciences, Peking University, Beijing 100191, China; 4 Xin’an Medical Education Ministry Key Laboratory, Hefei 230038, China

**Keywords:** Gandouling Pill, qualitatively analysis, absorbed components, quantitively analysis, alkaloids

## Abstract

Gandouling (GDL) Pill is a novel Traditional Chinese medicinal drug to treat Wilson’s disease in clinics. It is composed of six separate herbal medicines, including Rhei Radix ET Rhizoma, Coptidis Rhizoma, Salviae Miltiorrhizae Radix ET Rhizoma, Spatholobi Caulis, Curcumae Rhizoma, and Curcumae Longae Rhizoma. In this study, a strategy was proposed to investigate the chemical constituents and to quantify the potential bioactive components in GDL Pill. Firstly, the mass fragmentation behaviors of representative compounds were investigated, and, in total, 69 compounds were characterized in GDL Pill using full scan/dd-MS^2^ scan mode by ultra-high-performance liquid chromatography (UPLC)/Q-Orbitrap mass spectrometry (MS). These compounds included 18 alkaloids, 18 ketones, 16 phenolic compounds, 11 organic acids, and 6 tanshinones. Seventeen of the compounds were unambiguously identified by comparison with reference standards. Secondly, the absorption components of GDL Pill in rat plasma were investigated by using target-Selected Ion Monitoring (t-SIM) scan mode built in Q-Orbitrap MS. A total of 18 components were detected, which were considered as potential bioactive components of GDL Pill. Thirdly, 10 major absorption components were simultaneously determined in six batches of samples by UPLC/diode array detector (DAD). The method was fully validated with respect to linearity, precision, repeatability, stability, and recovery. Alkaloids from Coptidis Rhizoma, such as coptisine (**8**), berberine (**18**), palmatine (**19**), were the most abundant bioactive compounds for GDL Pill that possess the potential be used as quality markers. The proposed strategy is practical and efficient for revealing the material basis of GDL Pill, and also provides a simple and accurate method for quality control.

## 1. Introduction

Traditional Chinese medicines (TCMs) are always used in the form of formulae in clinical practice [1]. They are demonstrated to be “complex matrix” in structure with a large array of compounds [2,3,4,5]. Fully understanding the chemicals, especially the potentially bioactive ones, is vital for the safety and efficacy evaluation of TCM formulae. In the past decades, various analytical technologies were developed for TCM formulae, such as liquid chromatography/diode array detector (LC/DAD) and liquid chromatography/mass spectrometry (LC/MS) [6]. Among them, LC/MS is a cost-effective tool to characterize a large number of compounds from TCM formulae. In our previous report, a total of 259 compounds were rapidly detected and characterized in the Xiaoer–Feire–Kechuan formula [3]. Multi-components determination also plays a key role for quality control of TCM formulae. LC/DAD is a conventional technology, due to its strong applicability and easy operation. For example, by using LC/DAD method, a total of 19 compounds in the Xiaoer–Feire–Kechuan formula were simultaneously determined [5]. However, two major drawbacks emerged in the current quality evaluation for TCM formulae, as follow: (1) The chemical basis was not fully clarified; (2) Insufficient quality markers could not reflect the entirety of a formula. Obviously, it is imperative to develop more effective and comprehensive analytical methods to address the problems.

Wilson’s disease was first defined in 1912 as being caused by a copper metabolism disorder, which could also present with hepatic and neurological deficits, including dystonia and parkinsonism [7]. Gandouling (GDL) Pill is a novel TCM formula to treat Wilson’s disease by potentially improving liver function and cellular immune function, and combating cognitive and memory impairment and depression in patients [8,9,10]. It is composed of six separate component herbs, including Da-Huang (DH, Rhei Radix ET Rhizoma), Huang-Lian (HL, Coptidis Rhizoma), Dan-Shen (DS, Salviae Miltiorrhizae Radix ET Rhizoma), Ji-Xue-Teng (JXT, Spatholobi Caulis), E-Zhu (EZ, Curcumae Rhizoma), and Jiang-Huang (JH, Curcumae Longae Rhizoma). Hundreds of chemicals have been isolated from the single component herbs, mainly alkaloids, phenolic compounds, saponins, and organic acids, which show a wide range of acidity/alkalinity and polarity [11,12,13,14,15]. Although GDL Pill has been used in clinics to treat Wilson’s disease for a long time, the components in GDL Pill. let alone its potential bioactive components. have not been fully investigated till now. For example, only four compounds (berberine, coptisine, epiberberine, and palmatine) from HL were qualitatively and quantitatively analyzed using a LC/DAD method [16].

In this study, an integrated strategy was proposed to elucidate the chemical components in GDL Pill for the first time. Firstly, the chemical components of GDL Pill were investigated by using Full Scan/dd-MS^2^ scan mode built-in Q-Orbitrap MS. In total 69 compounds were characterized by verifying their MS and MS/MS spectra. Secondly, 18 absorption components of GDL Pill in rat plasma were detected using target-Selected Ion Monitoring (t-SIM) scan mode. Finally, 10 major absorption components were simultaneously determined in six batches of samples by LC/DAD. This study provides a simple and accurate method for quality control of GDL Pill.

## 2. Results

### 2.1. Optimization of the Extraction Method

According to the components of separate herbal medicines in GDL Pill, both hydrophilic compounds (e.g., alkaloids and flavonoid glycosides) and hydrophobic compounds (e.g., phenolic aglycones) may be involved. The extraction method was optimized to effectively extract both types of compounds. Different solvents (water, 50% methanol, 75% methanol, and methanol) were compared to fully extract the components in GDL Pill, and 75% methanol provided the best extraction efficiency for different types of compounds (Figure S1, Table S1). For example, the alkaloids 18/19 and phenolic aglycones 58/64 exhibited higher recovery in 75% methanol. Therefore, 75% methanol was chosen to extract the chemicals in GDL Pill.

### 2.2. Optimization of the Separation Method

Due to the rich alkaloids in HL, peak tailing is easily observed, which seriously influences the separation degree. Different types of stationary phases, including Acquity charged surface hybrid (CSH) C18 (2.1 × 100 mm, 1.7 μm, Waters, MA, USA), Acquity HSS T3 C18 (2.1 × 100 mm, 1.8 μm, Waters, MA, USA), Acquity Cortecs C18 (2.1 × 100 mm, 1.6 μm, Waters, MA, USA), SB-C18 column (2.1 mm × 150 mm, 1.8 µm, Agilent, MA, USA), were optimized using the real sample. As shown in Figure S2, the Acquity CSH C18 column provided favorable resolution for alkaloids (8–11, 18, 19), as well as for other compounds (41, 53, 58, 64). When comparing the peak shape using different types of mobile phases, it was illustrated that acidic additive was essential for baseline separation of alkaloids (Figure S3), and 0.1% formic acid in water was used for the following study.

### 2.3. Chemical Profiling of GDL Pill

**Figure 1:**
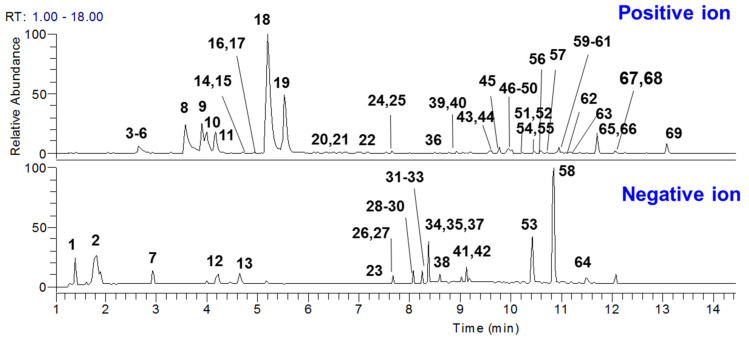
The LC/MS chromatograms of GDL Pill.

A high-resolution mass spectrometer was used to detect and identify the compounds in GDL Pill. In total 69 compounds were tentatively characterized, including 18 alkaloids, 16 phenolic compounds, 11 organic acids, 6 tanshinones, and 18 ketones (Table 1, Figure 1) [11,12,13,14,15,17,18,19]. Among them, 17 compounds were fully identified by comparing with reference standards. Moreover, by comparing with the MS spectra of separate herbs, the sources of characterized compounds were also identified (Figures S4 and S5). 

#### 2.3.1. Characterization of Alkaloids 

**Figure 2:**
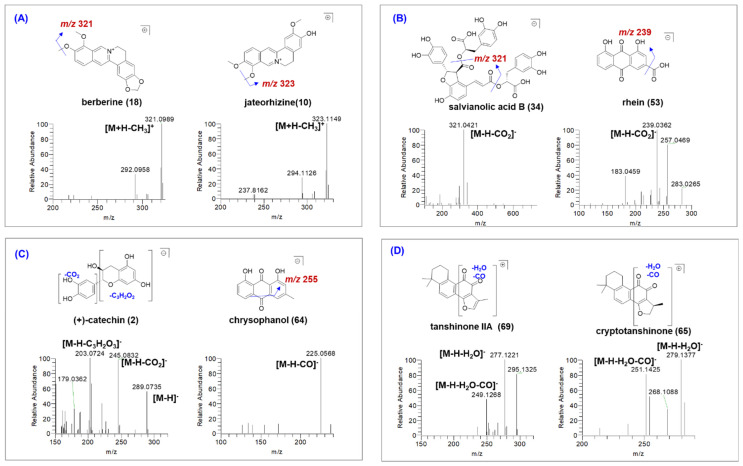
The MS/MS spectra of representative compounds identified in GDL Pill. (**A**) for alkaloids, (**B**) for organic acids, (**C**) for phenolic compounds, and (**D**) for tanshinones.

Alkaloids in GDL Pill are mainly from HL, which are easily ionized in positive ion mode. In this study, in total, 18 alkaloids were characterized in GDL Pill by comparing with standards or verifying their MS and MS/MS spectra. The alkaloids from HL usually contain methoxyl groups, and, thus, yield neutral loss (NL) of 15.0238 Da, corresponding to a methyl radical (CH_3_) in tandem mass spectrometry. For example, compounds **9** and **18** exhibited the [M + H]^+^ ions at *m*/*z* 336.12, and the corresponding molecular formula was C_20_H_18_O_4_N. In their MS/MS spectra, both of them yielded the product ion at *m*/*z* 321.09 [M+H-CH_3_]^+^ and *m*/*z* 292.10 [M+H-C_2_H_4_O]^+^. By comparing with reference standards, compounds 9 and 18 were respectively identified as epiberberine and berberine, by verifying their retention times, MS and MS/MS spectra (Figure 2A and Figure S6). Similarly, compounds **10** and **11** exhibited the [M + H]^+^ ions at *m*/*z* 323.09, and the corresponding molecular formula was C_20_H_20_O_4_N. In their MS/MS spectra, both of them also yielded the product ion at *m*/*z* 323.09 [M+H-CH_3_]^+^ and *m*/*z* 294.10 [M+H-C_2_H_4_O]^+^. By comparing with reference standards, compounds **10** and **11** were unambiguously identified as jateorhizine (Figure 2A) and columbamine (Figure S6), respectively. Compound **6** showed [M + H]^+^ ions at *m*/*z* 324.1235 (C_19_H_18_O_4_N). In the MS/MS spectrum, product ion at [M+H-CH_3_]^+^ was also observed (Figure S6). By comparing with literature, it was tentatively identified as demethyleneberberine [14]. 

#### 2.3.2. Characterization of Organic Acids

In total, 11 organic acids were characterized in GDL Pill, which were mainly from DS, HL, and DH. Due to the presence of carboxyl groups, organic acids are easily ionized in negative ion mode. For example, compound **34** exhibited the [M − H]^−^ ion at *m*/*z* 717.1466, and the corresponding molecular formula was C_36_H_29_O_16_. In the MS/MS spectra, compound **34** yielded the product ions at *m*/*z* 339.0526 [M−H-C_18_H_18_O_9_]^−^ and *m*/*z* 321.0421 [M−H-C_18_H_20_O_10_]^−^. By comparing with a reference standard, compound **34** was identified as salvianolic acid B by verifying the retention times, MS and MS/MS spectra (Figure 2B). Similarly, compound **53** exhibited the [M − H]^−^ ion at *m*/*z* 283.0626, and the corresponding molecular formula was C_15_H_7_O_6_. In the MS/MS spectra, it yielded the product ion at *m*/*z* 239.0362 [M−H-CO_2_]^−^, which demonstrated the presence of the carboxyl group. By comparing with a reference standard, compound **53** was unambiguously identified as rhein (Figure 2B). Compound **35** showed [M − H]^−^ ion at *m*/*z* 491.1012 (C_26_H_19_O_10_). In the MS/MS spectrum, product ions at *m*/*z* 311.0581 [M−H-C_9_H_8_O_4_]^−^ and *m*/*z* 293.0581 [M−H-C_9_H_10_O_5_]^−^ were observed (Figure S7). By comparing with literature, it was tentatively identified as salvianolic acid C. Compounds **7**, **12**, and **13** showed similar [M − H]^−^ ions at *m*/*z* 367.11 (C_17_H_19_O_9_). They were respectively characterized as 5-*O*-feruloylquinic acid, 3-*O*-feruloylquinic acid, and 4-*O*-feruloylquinic acid, according to their relative elution times when using a C18 reverse phase column (Figure S7) [12]. 

#### 2.3.3. Characterization of Phenolic Compounds

In total 16 phenolic compounds were characterized in GDL Pill, which were mainly from DH, JH, and JXT. Compounds **2** and **5** exhibited the [M − H]^−^ ions at *m*/*z* 289.07, and the corresponding molecular formula was C_15_H_13_O_6_. In their MS/MS spectra, both of them yielded the product ions at *m*/*z* 245.08 [M−H-CO_2_]^−^ and *m*/*z* 203.07 [M−H-C_3_H_2_O_3_]^−^. By comparing with reference standards, compounds **2** and **5** were, respectively, identified as (+)-catechin (Figure 2C) and epicatechin (Figure S8), by verifying the retention times, MS and MS/MS spectra. Similarly, compound **64** exhibited the [M − H]^−^ ion at *m*/*z* 253.0502, and the corresponding molecular formula was C_15_H_9_O_5_. In the MS/MS spectra, it yielded the product ion at *m*/*z* 225.0568 [M−H-CO]^−^. By comparing with reference standard, compound **64** was unambiguously identified as chrysophanol (Figure 2C). Compound **23** showed [M − H]^−^ ion at *m*/*z* 445.0800 (C_21_H_17_O_11_), which was 162 Da higher than rhein. In the MS/MS spectrum, product ions at *m*/*z* 283.0266 [M−H-Glc]^−^ were observed due to the breakage of the glucoside bond. By comparing with literature, this was tentatively identified as rhein-8-*O*-glucoside (Figure S8) [12]. Compound **38** showed the [M − H]^−^ ion at *m*/*z* 517.1014 (C_24_H_21_O_13_). In the MS/MS spectrum, product ion at *m*/*z* 269.0469 [M−H-Glc-malonyl]^−^ was observed. By comparing with literature, it was tentatively identified as malonyl-emodin-glucoside (Figure S8) [12]. 

#### 2.3.4. Characterization of Other Compounds

In total 6 tanshinones were characterized in GDL Pill. Due to the lack of hydroxyl group, tanshinones are not easily ionized in negative ion mode. Compound **69** exhibited the [M + H]^+^ ion at *m*/*z* 295.1332, and the corresponding molecular formula was C_15_H_9_O_5_. In the MS/MS spectra, it yielded the product ions at *m*/*z* 277.1221 [M−H-H_2_O]^−^ and *m*/*z* 249.1268 [M−H-H_2_O-CO]^−^. By comparing with literature, compound **69** was tentatively identified as tanshinone IIA (Figure 2D) [17]. Similarly, compound **65** was characterized as cryptotanshinone (Figure 2D) [18]. In addition, 18 ketones were also characterized in GDL Pill, and their structures were also tentatively characterized using a similar method (Table 1).

### 2.4. Absorption Components of GDL Pill in Rat Plasma

**Figure 3:**
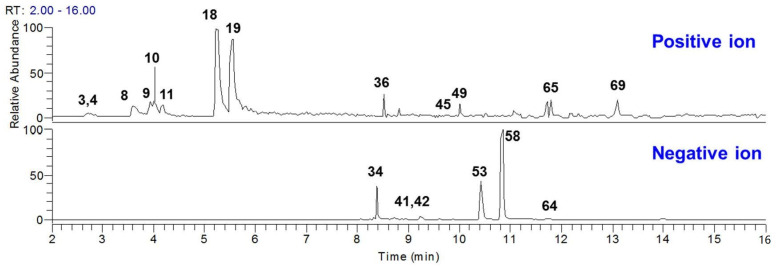
The t-SIM chromatograms of rat plasms after oral administration of GDL Pill.

**Figure 4:**
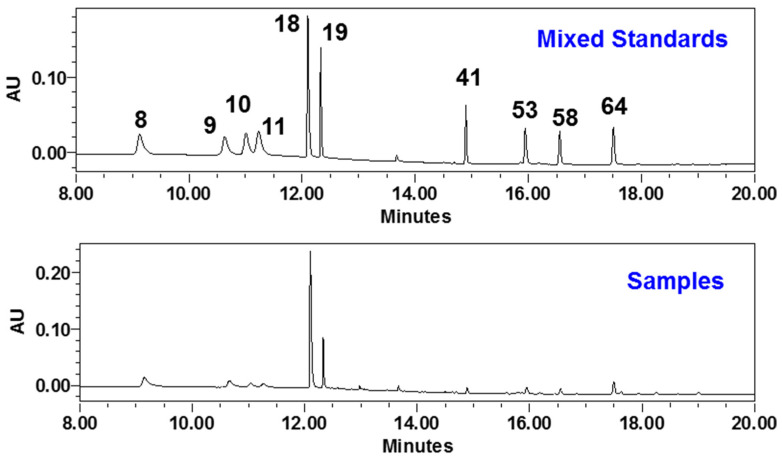
The LC/UV chromatograms of GDL Pill and mixed standards (270 nm).

Generally, the components that are absorbed in plasma after oral administration are always considered to be the bioactive ones for traditional Chinese medicines. Based on the chemical components that were characterized in GDL Pill, the plasma-absorption components were determined by a highly sensitive and selective targeted-selected reaction monitoring (t-SIM) scan mode when the GDL Pill was orally administered to rats. In total, 19 compounds were detected in rat plasma (Table 1, Figure 3). The extracted ion chromatograms of the 19 compounds are shown in Figure 4. These compounds included 9 alkaloids, 6 phenolic compounds, 2 organic acids, 2 tanshinones, and 1 ketone (Table 1). These compounds could be potential bioactive components of GDL Pill that could be used for quality control.

### 2.5. Quantitation of the Plasma-Absorption Components in GDL Pill

According to the investigation of drug metabolism of GDL Pill in rats, a total of 10 major compounds (coptisine-**8**, palmatine-**19**, berberine-**18**, epiberberine-**9**, jateorhizine-**11**, columbamine-**10**, chrysophanol-**64**, aloe-emodin-**41**, rhein-**53**, and emodin-**58**) were selected as quality markers for GDL Pill (Figure 4). Among them, six alkaloids (**8**–**11**, **18**, **19**, and **64**) were from HL, and six phenolic aglycones (**41**, **53**, **58**, and **64**) were from DH.

#### 2.5.1. Method Validation

The calibration curves of 10 analytes were constructed by plotting the analyte peak area (*y*) against the concentration (*x*). All the 10 analytes showed good linearity (*r*^2^ = 0.9973 − 1.0) (Table 2). The stability was evaluated by analyzing the same sample solution at 0, 2, 4, 8, 12, and 24 h at room temperature (25 ± 2 °C). The RSD values for stability analysis ranged from 0.45% to 4.41%. The precision of the method was evaluated by analyzing the same reference solution six times continuously (intra-day) in the following three days (inter-day). The RSD values for intra-day and inter-day precisions ranged from 0.12% to 1.62% and 0.43% to 1.96%, respectively, indicating acceptable precision of the method. The repeatability was evaluated by injecting six independently prepared sample solutions. The reproducibility test showed a good consistency of the sample preparation process with RSD values ranging from 0.42%–4.26%. The accuracy was measured by spiking the reference standards at 100% level (equivalent to the concentrations in the sample solution) into sample solutions (*n* = 6). Recovery of the analytes varied from 96.4% to 106.2%, indicating acceptable accuracy of this method.

#### 2.5.2. Sample Analysis

**Figure 5:**
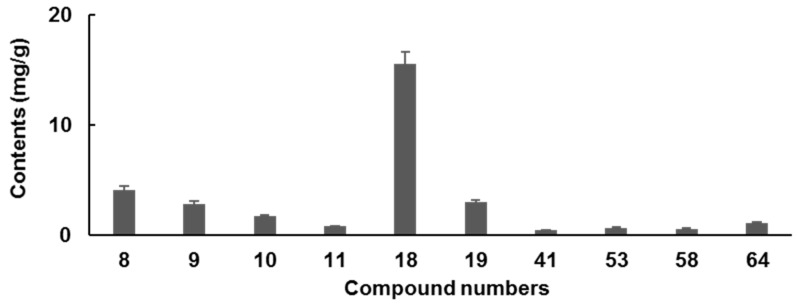
The contents of 10 potential bioactive components in GDL Pill (*n* = 6).

Contents of 10 potential bioactive compounds in 6 batches of GDL Pill were determined (Figure 5). The total contents of these 10 compounds varied from 29.54 to 31.10 mg/g, suggesting good quality consistency. Alkaloids were the major components in GDL Pill with contents at 28.19 ± 1.41 mg/g. Among these, six alkaloids, berberine (**18**) and coptisine (**8**) were the predominant constituents. Their contents among the 6 batches of samples were also similar, i.e., 15.54 ± 0.78 mg/g for 18 and 4.16 ± 0.21 mg/g for **8**. For 4 phenolic aglycones, chrysophanol (**64**) was the most abundant one, the contents of which varied from 1.11 to 1.22 mg/g. The total content of the other 3 phenolic aglycones (**41**, **53**, **58**) was 2.91 ± 0.15 mg/g. 

## 3. Materials and Methods

### 3.1. Chemicals and Reagents

**Figure 6:**
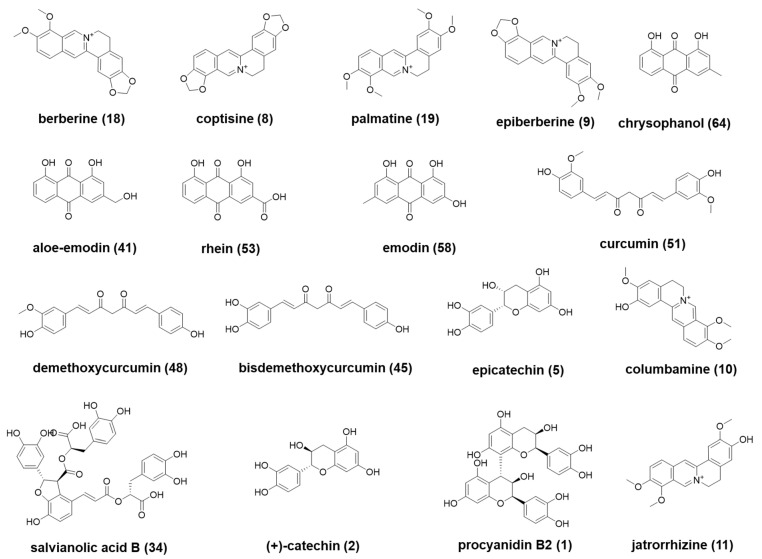
Structures of 17 reference compounds used in this study.

The reference standards of berberine (**18**), coptisine (**8**), palmatine (**19**), jatrorrhizine (**11**), chrysophanol (**64**), curcumin (**51**), demethoxycurcumin (**48**), and bisdemethoxycurcumin (**45**) were purchased from Chengdu DeSiTe Biological Technology Co., Ltd. (Chengdu, China). Columbamine (**10**), salvianolic acid B (**34**), epiberberine (**9**), aloe-emodin (**41**), rhein (**53**), emodin (**58**), (+)-catechin (**2**), procyanidin B2 (**1**), and epicatechin (**5**) were purchased from Chengdu MUST Biological Technology Co., Ltd. (Chengdu, China). Their structures are shown in Figure 6. Their purities were > 98% by HPLC analysis. HPLC grade methanol, acetonitrile, and formic acid were obtained from Fisher Scientific (Branchburg, NJ, USA). De-ionized water was prepared by Milli-Q purification system (Millipore, MA, USA).

Separate herbs, including Dahuang (DH, Rhei Radix ET Rhizoma), Huanglian (HL, Coptidis Rhizoma), Danshen (DS, Salviae Miltiorrhizae Radix ET Rhizoma), Jixueteng (JXT, Spatholobi Caulis), Ezhu (EZ, Curcumae Rhizoma), and Jianghuang (JH, Curcumae Longae Rhizoma), and GDL Pill (batch 1–6) were kindly donated by Anhui University of Chinese Medicine. Voucher specimens were deposited at the Anhui University of Chinese Medicine (Anhui, China).

### 3.2. Sample Solution Preparation

#### 3.2.1. Preparation of Reference Standard Solutions

For qualitative analysis, an appropriate amount of the 17 reference standards was dissolved in 75% methanol (*v*/*v*) to prepare a mixed standard solution (10.0 μg/mL for each compound). For quantitative analysis, a mixed stock solution was prepared by dissolving appropriate amounts of each reference standard in 75% methanol (*v*/*v*) at 1.0 mg/mL. The mixed standard solution was obtained by adding 200 μL of berberine (**18**), 100 μL of coptisine (**8**), palmatine (**19**), epiberberine (**9**), jateorhizine (**11**), columbamine (**10**), and 50 μL of chrysophanol (**64**), aloe-emodin (41), rhein (**53**), emodin (**58**) stock solutions to a 1 mL volumetric flask. The mixed standard solution was then serially diluted (dilution factor = 2, 4, 8, 16, 32, and 64) using 75% methanol (*v*/*v*).

#### 3.2.2. Preparation of Sample Solutions

For qualitative analysis, 200 mg of GDL Pill extracted in 20 mL of 75% methanol (*v*/*v*) for 30 min in an ultrasonic water bath (40 kHz, 500 W). Accurately, 300 mg of the HL, DH, DS, JXT, EZ, and JH powders were, respectively, extracted with 30 mL of 50% methanol (*v*/*v*) for 30 min in an ultrasonic water bath (40 kHz, 500 W). For quantitative analysis, 50 mg of GDL Pill extracted in 20 mL of 75% methanol (*v*/*v*) for 30 min in an ultrasonic water bath (40 kHz, 500 W).

### 3.3. Animal Experiments

Eight male SD rats weighing 220 ± 20 g were obtained from Beijing Weitong Lihua Experimental Animals Company (Beijing, China). The rats were housed in a controlled room at standard temperature (24 ± 2°C) and humidity (70 ± 5%), and kept on a 12 h light/12 h dark regime. After a week acclimation, rats were randomly divided into two groups: Drug Group (*n* = 4) for test plasma; Control Group (*n* = 4) for blank plasma. They were fasted for 12 h with free access to water prior to the experiment. The animal protocols were approved by the institutional Animal Care and Use Committee at Anhui University of Chinese Medicine.

GDL Pill was suspended in 0.5% carboxymethylcellulose sodium (CMC-Na) solution. Rats in Drug Group were given a dose of 77.15 mg/kg body weight orally (equivalent to clinical dosage). 0.5% CMC-Na aqueous solution (2 mL) was administrated to rats in Control Group. Blood samples (0.5 mL) were taken from the suborbital venous plexus of rats at 0.5, 1, 2 and 4 h post-administration. All homogeneous biological samples from the same group were merged into a collective sample.

### 3.4. Liquid Chromatography

For qualitative analysis, a Vanquish UHPLC system (Thermo Fisher Scientific Inc., Waltham, MA, USA) was used. Samples were separated on an Acquity CSH column (2.1 × 100 mm,1.7 μm, Waters, MA, USA). The mobile phase A was water containing 0.1% formic acid and B was acetonitrile. The gradient elution program was set as follows: 0–4 min, 10%–25% B; 4–8 min, 25%–35% B; 8–16 min, 35%–45% B; 16–20 min, 45%–75% B; 20–22 min, 75%–95% B; 22–24 min, 95%B. The flow rate was 300 μL/min and the column temperature was set at 40 °C. The injection volume was 2 μL. For quantitative analysis, the stationary and mobile phases were the same as for qualitative analysis. The gradient elution program was set as follows: 0 min, 5% B; 10 min, 12% B; 14 min, 50% B; 21 min, 80% B. The flow rate was 400 L/min and the column temperature was set at 50 °C. The UV wavelength was 270 nm. The injection volume was 2 μL.

### 3.5. Mass Spectrometry 

Mass spectrometry analysis was performed on a Q-Exactive Plus hybrid quadrupole Orbitrap mass spectrometer (Thermo Scientific, San Jose, CA, USA) equipped with a heated electrospray ionization source (HESI). It was operated in both negative and positive ion modes. The other parameters were set as follows: spray voltage, ±3.5 kV; sheath gas flow rate, 35 arb; auxiliary gas, 10 arb; capillary temperature, 350 °C; auxiliary temperature, 400 °C; S-lens RF level, 60 V. Full Scan/dd-MS^2^ was used to acquire the qualitative data. The resolution for MS and MS/MS was set as 70,000 and 17,500, respectively. The scan range was set as *m*/*z* 100–1500, and the normalized collision energies (NCE) were 35%. The five most abundant ions in each full scan were selected as precursor ions to obtain their MS/MS spectra. Data were processed using Xcalibur^TM^ 4.1 software (Thermo Fisher). For t-SIM scan mode, the accurate [M − H]^−^ or [M + H]^+^ of detected compounds in GDL Pill was added in the Inclusion List to increase the detection sensitivity.

## 4. Conclusions

In this study, an integrated strategy was proposed to reveal the chemical components for GDL Pill. Firstly, 69 compounds were characterized using Full Scan/dd-MS^2^ scan mode built-in Q-Orbitrap MS, and 17 of them were unambiguously determined by comparison with reference standards. Secondly, 18 plasma-absorbed components were detected using t-SIM scan mode, which were considered to be potential bioactive components for GDL Pill. Finally, the contents of 10 major absorption components were simultaneously determined in six batches of samples by the UPLC/DAD method. Alkaloids from Coptidis Rhizoma, including coptisine (**8**), berberine (**18**), and palmatine (**19**), were the most abundant bioactive compounds for GDL Pill that could be used as quality markers. The established method is practical and efficient for the quality control of GDL Pill.

## Figures and Tables

**Table 1:** Characterization of chemical constituents in GDL Pill by HRMS/MS data.

Peak	*t_R_*	Formula	Measured [M − H]^−^/[M + H]^+^ (*m*/*z*)	Error (ppm)	Ion Mode	MS/MS Fragments	Source	Identification	Type	Plasma
1 *	1.53	C_30_H_25_O_12_	577.1389	2.4	−	407.0789, 289.0739, 125.0251	JXT	procyanidin B2 [11]	phenolic	
2 *	1.88	C_15_H_13_O_6_	289.0735	1.3	−	245.0831, 203.0724, 109.0300	DH	(+)-catechin [12]	phenolic	
3	2.62	C_19_H_18_NO_4_	324.1235	0.7	+	309.0989	HL	demethyleneberberine [13]	alkaloid	+
4	2.65	C_19_H_16_NO_4_	322.1077	1.3	+	307.0838, 294.1135, 279.0910	HL	thalifendine or groenlandicine [13]	alkaloid	+
5 *	2.68	C_15_H_13_O_6_	289.0736	2.3	−	245.0830, 203.0725, 109.0300	JXT	epicatechin [11]	phenolic	
6	2.76	C_19_H_18_NO_4_	324.1231	0.3	+	309.0989	HL	demethyleneberberine/isomer [13]	alkaloid	
7	2.90	C_17_H_19_O_9_	367.1053	3.2	−	193.0515, 134.0380	HL	5-*O*-feruloylquinic acid [14]	organic acid	
8 *	3.56	C_19_H_14_NO_4_	320.0921	1.4	+	292.0968, 236.8748	HL	coptisine [13]	alkaloid	+
9 *	3.89	C_20_H_18_NO_4_	336.1233	0.9	+	320.0908, 308.1270, 292.0979	HL	epiberberine [13]	alkaloid	+
10 *	4.00	C_20_H_20_NO_4_	338.1387	0.7	+	323.1150, 308.0917, 294.1116	HL	columbamine [13]	alkaloid	+
11 *	4.16	C_20_H_20_NO_4_	338.1389	0.9	+	323.1145, 308.0905, 294.1126	HL	jatrorrhizine [13]	alkaloid	+
12	4.17	C_17_H_19_O_9_	367.1052	4.2	−	193.0514, 173.0463	HL	3-*O*-feruloylquinic acid [14]	organic acid	
13	4.63	C_17_H_19_O_9_	367.1054	4.3	−	191.0571, 173.0463	HL	4-*O*-feruloylquinic acid [14]	organic acid	
14	4.66	C_36_H_38_NO_12_	676.2398	1.4	+	430.4002, 334.1067	HL	coptichine-quinic acid conjungate-CO + 2H [13]	alkaloid	
15	4.69	C_20_H_16_NO_4_	334.1078	1.3	+	306.1124	HL	worenine [13]	alkaloid	
16	4.91	C_21_H_20_NO_4_	350.1390	1.8	+	334.1051	HL	worenine + CH2 + 2H [13]	alkaloid	
17	5.07	C_36_H_38_NO_12_	676.2398	1.4	+	430.4001, 334.1066	HL	coptichine-quinic [13] acid conjungate-CO + 2H # [13]	alkaloid	
18 *	5.18	C_20_H_18_NO_4_	336.1234	1.0	+	321.0989, 292.0956	HL	berberine [13]	alkaloid	+
19 *	5.51	C_21_H_22_NO_4_	352.1546	0.8	+	337.1306, 322.1067, 308.1273	HL	palmatine [13]	alkaloid	+
20	6.34	C_21_H_20_NO_4_	350.1392	1.5	+	335.1153, 306.1127	HL	worenine + CH2 + 2H [13]	alkaloid	
21	6.59	C_30_H_26_NO_8_	528.1666	−0.8	+	334.1072, 319.0836	HL	demethylcoptichine/isomer [13]	alkaloid	
22	7.17	C_15_H_21_O_2_	233.1540	1.8	+	175.1120	EZ	furanogermenone [15]	ketone	
23	7.48	C_21_H_17_O_11_	445.0800	1.8	−	283.0266, 239.0362	DH	rhein-8-glucoside [12]	phenolic	
24	7.64	C_30_H_26_NO_8_	528.1663	−1.5	+	334.1071, 319.0834	HL	demethylcoptichine/isomer [13]	alkaloid	
25	7.64	C_31_H_28_NO_9_	558.1763	0.9	+	334.1069, 319.0836	HL	coptichine + O [13]	alkaloid	
26	7.68	C_22_H_21_O_11_	461.1118	1.5	−	313.0581, 169.0150, 147.0458	DH	rumejaposide D [12]	phenolic	
27	7.68	C_38_H_17_O_4_	537.1077	−3.2	−	339.0527, 295.0626, 185.0252	DS	lithospermic acid [17]	organic acid	
28	8.08	C_22_H_19_O_12_	475.0883	1.8	−	269.0469	DH	endocrocin-glucoside [12]	phenolic	
29	8.10	C_38_H_17_O_4_	537.1071	−3.6	−	295.0622, 185.0254, 109.0299	DS	lithospermic acid/isomer [6]	organic acid	
30	8.10	C_26_H_21_O_10_	493.1167	4.3	−	295.0625, 185.0252, 109.0300	DS	salvianolic acid A [17]	organic acid	
31	8.24	C_14_H_23_O_15_	431.1007	−2.3	−	268.0391	DH	aloe-emodin-1-glucoside/isomer [12]	phenolic	
32	8.27	C_26_H_21_O_10_	493.1169	3.4	−	295.0625, 185.0252, 109.0300	DS	salvianolic acid A/isomer [17]	organic acid	
33	8.28	C_14_H_23_O_15_	431.1008	−2.4	−	269.0470	DH	aloe-emodin-1-glucoside/isomer [12]	phenolic	
34 *	8.38	C_36_H_29_O_16_	717.1504	4.3	−	339.0526, 321.0421, 295.0629, 109.0301	DS	salvianolic acid B [17]	organic acid	+
35	8.46	C_26_H_19_O_10_	491.1012	−3.7	−	311.0581, 293.0473, 135.0459	DS	salvianolic acid C [17]	organic acid	
36	8.51	C_20_H_16_NO_7_	382.0928	1.8	+	318.0754, 190.0499	HL	dehydro-chilenine [13]	alkaloid	+
37	8.51	C_22_H_19_O_11_	459.0959	3.2	−	266.0597, 253.0519	DH	2-carboxyl chrysophanol-glc I [12]	phenolic	
38	8.84	C_24_H_21_O_13_	517.1014	2.6	−	269.0470	DH	malonyl-emodin-glucoside [12]	phenolic	
39	8.92	C_15_H_19_O_3_	247.1330	0.8	+	139.0391, 123.0443	EZ	zederone/isomer [15]	ketone	
40	8.92	C_15_H_23_O_2_	235.1697	2.2	+	189.1637, 177.1275	EZ	curcumenone/isomer [15]	ketone	
41 *	9.24	C_15_H_9_O_5_	269.0470	4.3	−	240.0440	DH	aloe-emodin [12]	phenolic	
42	9.27	C_18_H_13_O_8_	357.0636	2.3	−	225.0569, 181.0670, 121.0301	DS	salvianic acid C [17]	organic acid	+
43	9.78	C_15_H_23_O	217.1588	0.6	+	161.0957	EZ	furanodiene/isomer [15]	ketone	
44	9.78	C_15_H_23_O_2_	235.1695	1.2	+	177.1272, 161.0959	EZ	curcumenone/isomer [15]	ketone	
45 *	9.88	C_19_H_17_O_6_	309.1123	0.8	+	225.0910, 147.0441	JH	bisdemethoxycurcumin [18]	phenolic	+
46	9.95	C_15_H_23_O	217.1589	1.3	+	161.0964	EZ	furanodiene/isomer [15]	ketone	
47	9.95	C_15_H_23_O_2_	235.1695	1.2	+	189.1639, 161.0963	EZ	Curcumenol [15]	ketone	
48 *	10.00	C_20_H_19_O_6_	339.1232	1.7	+	255.1016, 177.0547, 147.0441	JH	demethoxycurcumin [18]	phenolic	
49	10.03	C_15_H_19_O_3_	247.1330	0.8	+	139.0390, 123.0444	EZ	zederone [15]	ketone	+
50	10.03	C_15_H_17_O_2_	229.1225	1.2	+	201.1274, 123.0443	EZ	curzeone/isomer [15]	ketone	
51 *	10.11	C_21_H_21_O_6_	369.1338	1.6	+	285.1125, 253.0859, 177.0547	JH	curcumin [18]	phenolic	
52	10.22	C_15_H_25_O_2_	237.1852	1.4	+	219.1746, 135.1169	EZ	Neocurdione [15]	ketone	
53 *	10.41	C_15_H_7_O_6_	283.0262	3.5	−	257.0469, 239.0362	DH	rhein [12]	organic acid	+
54	10.45	C_15_H_25_O_2_	237.1852	1.4	+	219.1741, 135.1169	EZ	curdione [15]	ketone	
55	10.45	C_15_H_23_O	219.1746	1.3	+	135.1170	EZ	germacrone/isomer [15]	ketone	
56	10.58	C_18_H_15_O_3_	279.1020	1.8	+	261.0909, 233.0961, 205.1009	DS	dihydrotanshinone I [19]	tanshinone	
57	10.70	C_15_H_17_O_2_	229.1226	1.3	+	201.1274	EZ	curzeone/isomer [15]	ketone	
58 *	10.83	C_15_H_9_O_5_	269.0469	4.2	−	241.0518, 225.0569	DH	emodin [12]	phenolic	+
59	10.89	C_18_H_17_O_3_	281.1174	0.9	+	263.1065, 235.1116	DS	danshenxinkun B [19]	tanshinone	
60	10.95	C_15_H_17_O	213.1275	0.9	+	198.1042, 185.1320	EZ	Pyrocurzerenone [15]	ketone	
61	10.95	C_15_H_19_O_2_	231.1382	1.4	+	213.1267, 173.0959, 83.0862	EZ	curzerenone/isomer [15]	ketone	
62	11.17	C_15_H_19_O_2_	231.1382	1.4	+	213.1279, 83.0860	EZ	curzerenone/isomer [15]	ketone	
63	11.31	C_15_H_19_O_2_	231.1383	1.7	+	213.1273, 203.1432	EZ	curzerenone/isomer [15]	ketone	
64 *	11.71	C_15_H_9_O_4_	253.0519	3.2	−	225.0568	DH	chrysophanol [12]	phenolic	+
65	11.75	C_19_H_21_O_3_	297.1488	1.0	+	279.1377, 251.1425	DS	cryptotanshinone [19]	tanshinone	+
66	11.75	C_18_H_13_O_3_	277.0860	0.3	+	249.0904	DS	tanshinone I [19]	tanshinone	
67	12.37	C_15_H_23_O	219.1747	1.5	+	135.1167	EZ	germacrone/isomer [15]	ketone	
68	12.43	C_19_H_17_O_3_	293.1174	0.8	+	275.1057, 247.1114	DS	1,2 -didehydrotanshinone IIA [19]	tanshinone	
69	13.08	C_19_H_19_O_3_	295.1332	1.4	+	277.1221, 249.1268	DS	tanshinone IIA [19]	tanshinone	+

JXT: Ji-Xue-Teng, Spatholobi Caulis; DH: Da-Huang, Rhei Radix ET Rhizoma; HL: Huang-Lian, Coptidis Rhizoma; DS: Dan-Shen, Salviae Miltiorrhizae Radix ET Rhizoma; EZ: E-Zhu, Curcumae Rhizoma; JH: Jiang-Huang, Curcumae Longae Rhizoma; * confirmed by reference standard.

**Table 2:** Method validation results for quantitative analysis of 10 compounds in GDL Pill.

Analytes	Regression Equations	*r* ^2^	Linear Range (μg/mL)	Precious		Repeatability (*n* = 6)	Stability (*n* = 6)	Recovery (*n* = 6)			
				Intra-Day (*n* = 6)	Inter-Day (*n* = 3)			Spiked (μg)	Found (μg)	Recovery (%)	RSD (%)
coptisine (**8**)	*y* = 184,80*x* − 1005.9	0.9999	1.56–25.0	0.12	0.52	1.77	0.60	2.43	2.50	97.06	1.24
epiberberine (**9**)	*y* = 16,328*x* − 2759.6	0.9992	1.56–25.0	0.45	0.59	4.26	4.41	2.45	2.50	97.82	1.26
columbamine (**10**)	*y* = 17,745*x* + 1440	0.9995	1.56–25.0	0.42	0.43	2.48	4.05	2.41	2.50	96.37	1.34
jateorhizine (**11**)	*y* = 25,348*x* + 37,911	0.9973	1.56–25.0	1.03	1.96	3.57	2.01	2.59	2.50	103.43	1.69
berberine (**18**)	*y* = 18,120*x* − 1359.4	0.9995	3.13–50.0	0.29	0.79	3.22	2.24	4.74	5.00	94.84	2.24
palmatine (**19**)	*y* = 20,530*x* + 39,866	1.0000	1.56–25.0	1.62	1.33	3.34	2.33	2.66	2.50	106.21	1.70
aloe-emodin (**41**)	*y* = 10,137*x* + 1241.4	0.9996	0.78–12.5	0.22	0.81	3.13	3.90	1.24	1.25	98.96	1.14
rhein (**53**)	*y* = 14,615*x* + 3046.5	0.9993	0.78–12.5	0.32	1.63	1.07	1.22	1.21	1.25	96.58	0.99
emodin (**58**)	*y* = 17,564*x* + 889.7	0.9999	0.78–12.5	0.34	0.61	0.99	1.45	1.23	1.25	98.04	1.81
chrysophanol (**64**)	*y* = 11,515*x* + 1484.2	0.9997	0.78–12.5	0.74	*0.60*	0.42	0.45	1.23	1.25	98.62	4.81

## Data Availability

No data available.

## Supplementary Materials

The following supporting information can be downloaded at: https://www.mdpi.com/article/10.3390/molecules27238247/s1, Figure S1: Extraction efficiency of compounds in GDL Pills using different solvents; Figure S2: Separation efficiency of compounds in GDL Pills using different stationary phases; Figure S3: Separation efficiency of compounds in GDL Pills using different mobile phases; Figure S4: The LC/MS chromatograms of GDL Pill and separate herbs in the positive ion mode; Figure S5: The LC/MS chromatograms of GDL Pill and separate herbs in the negative ion mode; Figure S6: The MS/MS spectra of representative alkaloids identified in GDL Pill; Figure S7: The MS/MS spectra of representative organic acids identified in GDL Pill; Figure S8: The MS/MS spectra of representative phenolics identified in GDL Pill; Table S1 Comparison of peak areas of 6 major compounds in GDL Pill by using different kinds of extraction solvent.

## Sample Availability

Samples of the compounds are not available from authors.
