# Supplementary material for: Chemical Profiling and Quantification of Potential Bioactive Components in Gandouling Pill by Ultra-High Performance Liquid Chromatography Coupled with Diode Array Detector/Quadruple-Qrbitrap Mass Spectrometry
Source: Molecules. 2022 Nov 26;27(23):8247. doi: 10.3390/molecules27238247 (PMC9740684; doi:10.3390/molecules27238247)
Supplement: Supplementary file 1 [file molecules-27-08247-s001.zip › molecules-2015802-supplementary.pdf]

Chemical profiling and quantification of potential bio-active components in Gandouling Pills by ultra-high performance liquid chromatography coupled with high quadruple-Qrbitrap mass spectrometry

Yue Yang[a], Wenjie Hao[a], Yulong Yang[a], Shijie Zhang[b], Han Wang[b], Meixia Wang[b], Ting Dong[b], Zhanpeng Shang[c,*], Wenming Yang[b,d,*]

[a] *Graduate School of Anhui University of Chinese Medicine, Hefei, P.R. China*

[b] *The First Affifiliated Hospital of Anhui University of Chinese Medicine, Hefei, P.R. China*

[c] *School of Pharmaceutical Sciences, Peking University, Beijing 100191, P.R. China*

[d] *Xin'an Medical Education Ministry Key Laboratory, Hefei, P.R. China*

* Corresponding author

E-mail address: Wenming Yang (yangwm8810@126.com) and Zhanpeng Shang (zpshang1206@163.com)

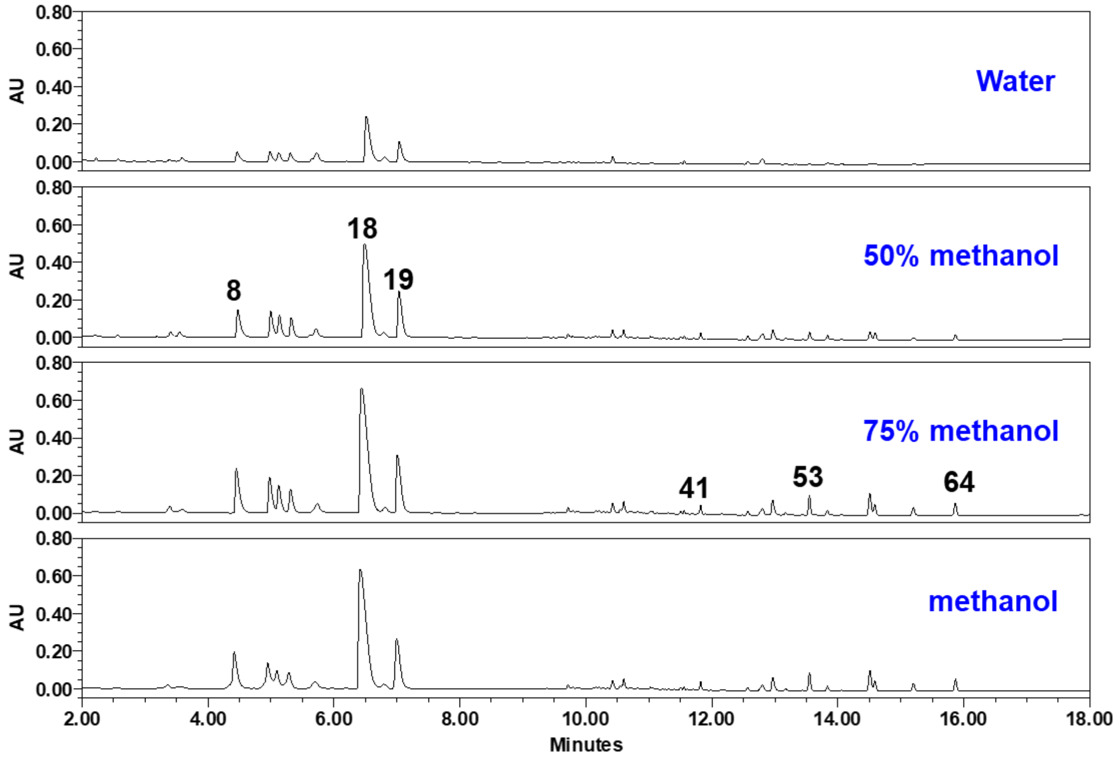

Figure S1 Extraction efficiency of compounds in GDL Pills using different solvents (270 nm).

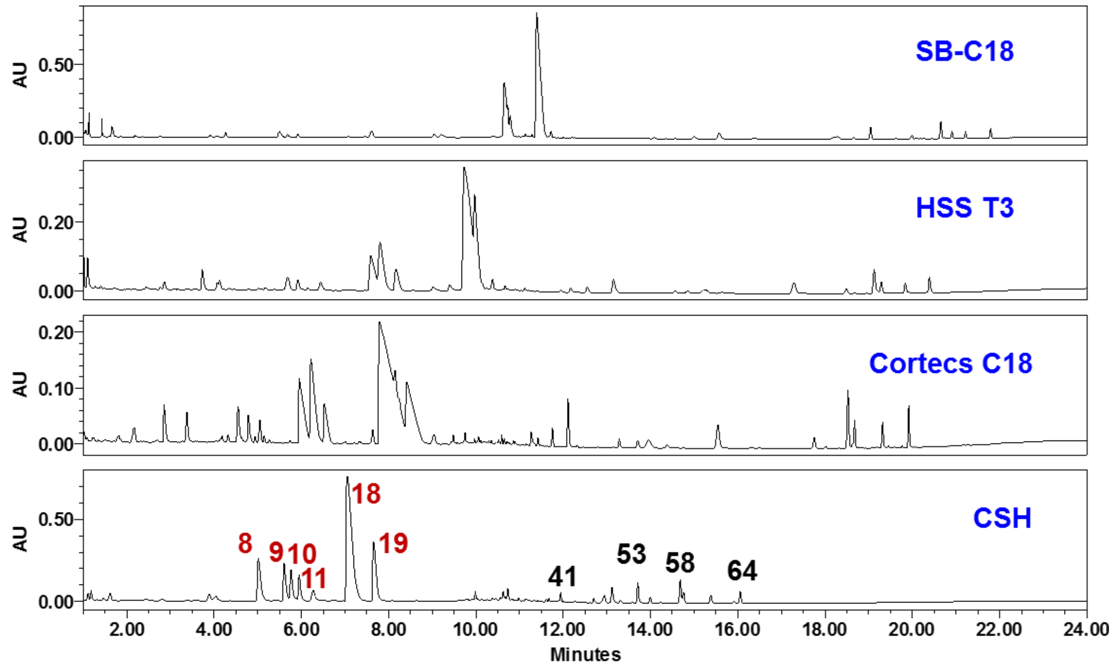

Figure S2 Separation efficiency of compounds in GDL Pills using different stationary phases. The alkaloids were marked in red and other compounds marked in black (270 nm).

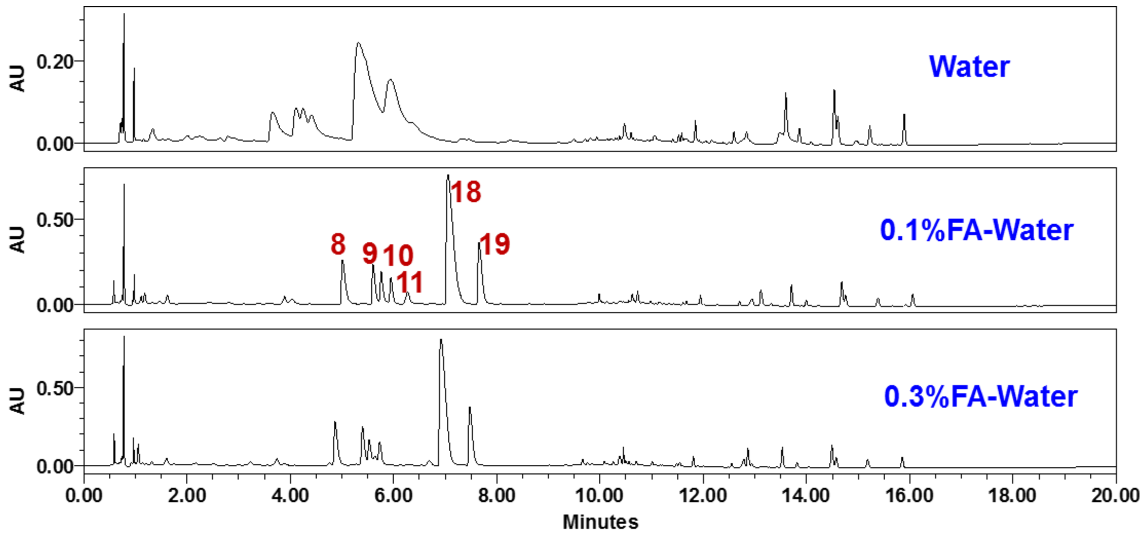

Figure S3 Separation efficiency of compounds in GDL Pills using different mobile phases (270 nm).

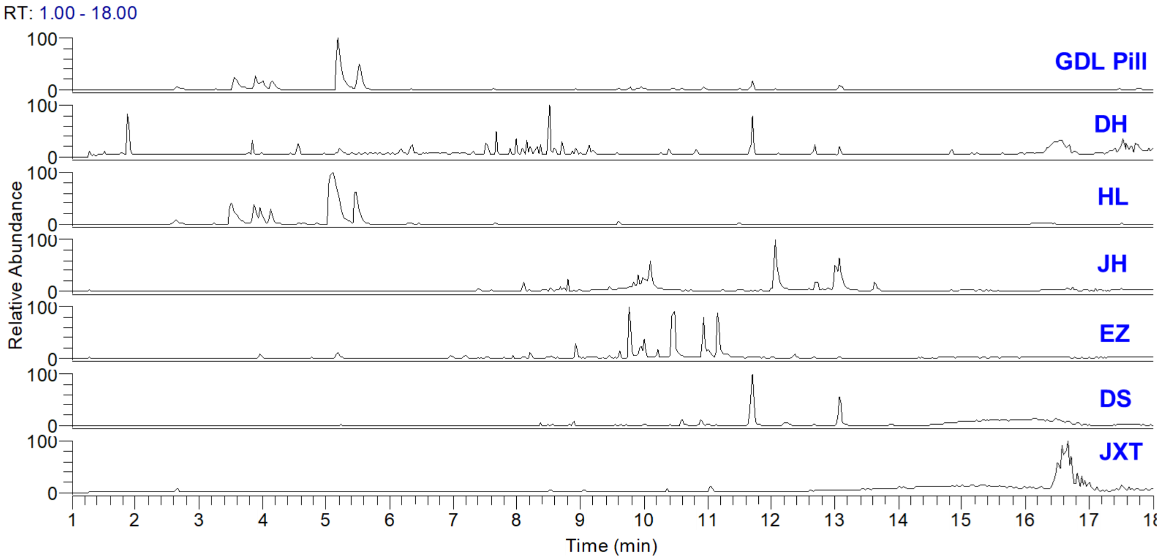

Figure S4 The LC/MS chromatograms of GDL Pill and separate herbs in the positive ion mode.

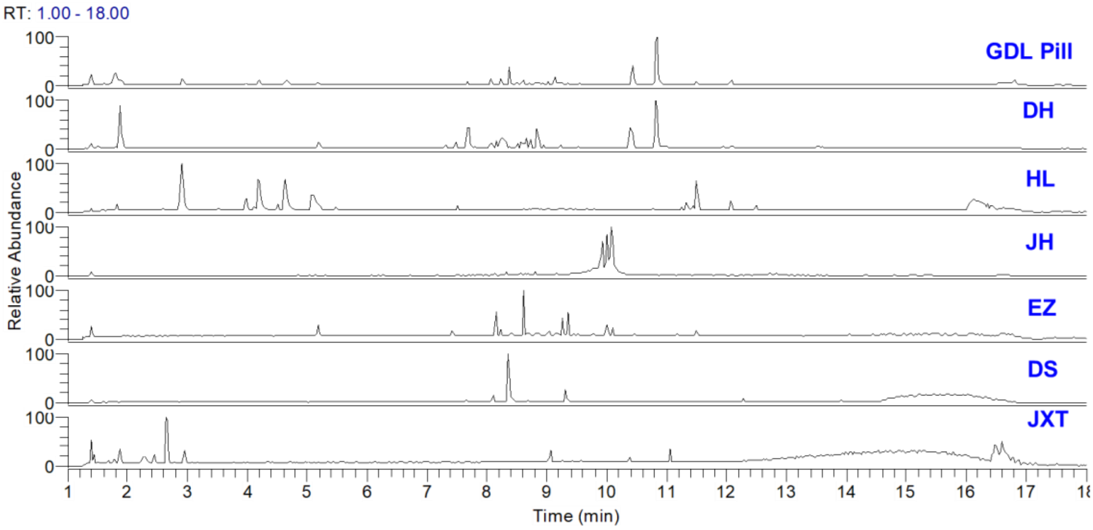

Figure S5 The LC/MS chromatograms of GDL Pill and separate herbs in the negative ion mode.

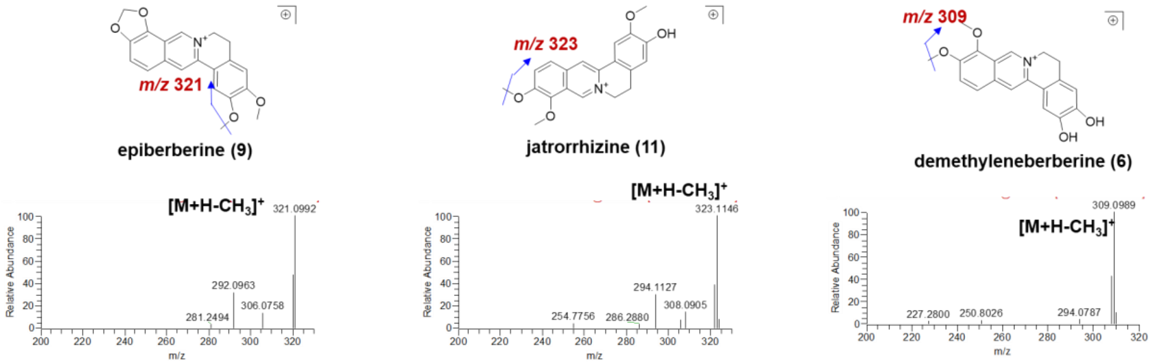

Figure S6 The MS/MS spectra of representative alkaloids identified in GDL Pill.

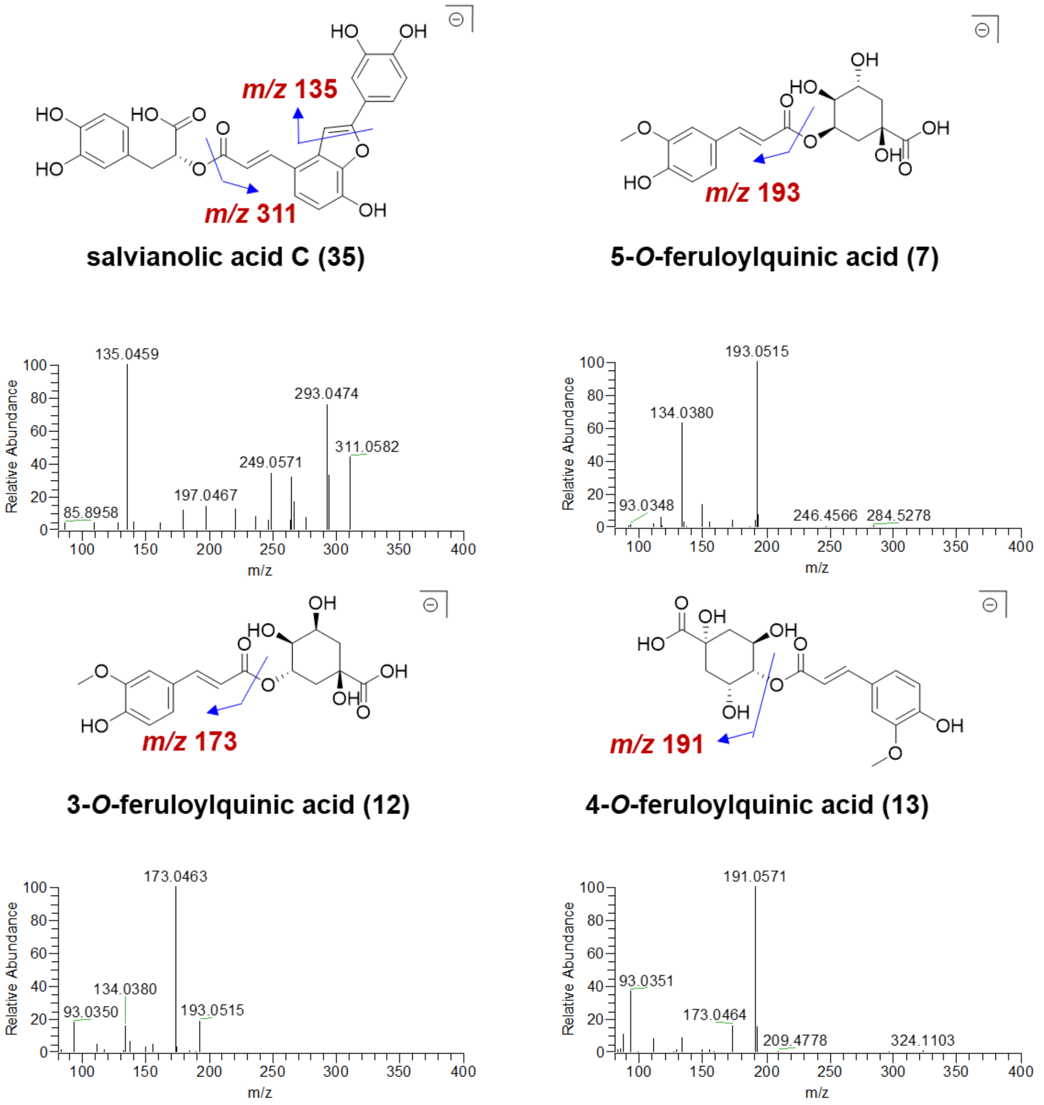

Figure S7 The MS/MS spectra of representative organic acids identified in GDL Pill.

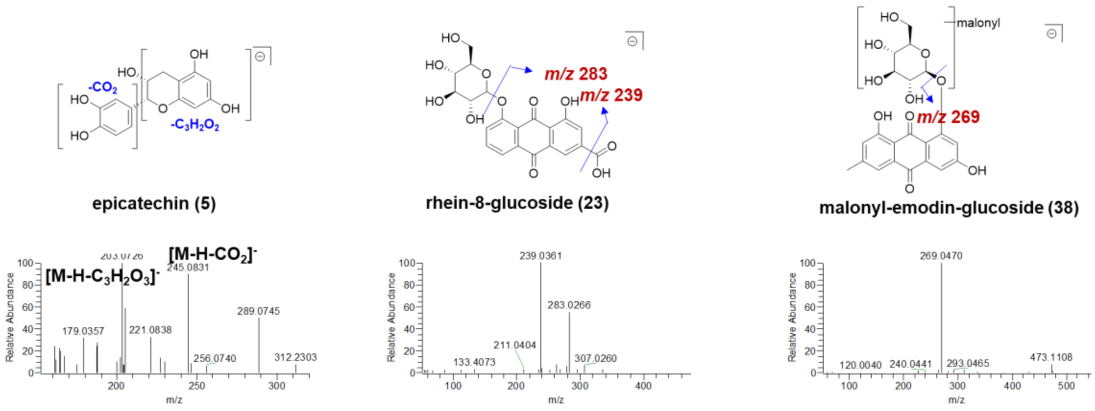

Figure S8 The MS/MS spectra of representative phenolics identified in GDL Pill.

Table S1 Comparison of extraction recovery (%) of 6 major compounds in GDL Pill by using different kinds of extraction solvent.

Peaks	water	50% methanol	75% methanol	methanol
8	75.06	92.31	100.00	99.00
18	72.95	89.42	100.00	98.22
19	68.42	84.37	99.87	100.00
41	55.61	83.31	100.00	96.55
53	57.48	86.74	100.00	97.77
64	57.69	81.23	99.45	100.00
